# Personal Metaphors as Motivational Resources: Boosting Anticipated Incentives and Feelings of Vitality Through a Personal Motto-Goal

**DOI:** 10.3389/fpsyg.2021.566215

**Published:** 2021-04-13

**Authors:** Thomas H. Dyllick, Oliver Dickhäuser, Dagmar Stahlberg

**Affiliations:** Department of Psychology, University of Mannheim, Mannheim, Germany

**Keywords:** motto-goals, activity related incentives, vitality, metaphors, motivation, autonomy

## Abstract

Motto-goals describe a desired mind-set and provide a person with a guiding principle of how to approach a personal goal or obligation (e.g., with the inner strength of a bear I am forging ahead). We propose that motto-goals can be conceptionalized as individually created metaphors and that the figurative, metaphorical language and the characteristics of the formation process make them effective in changing the perception of unpleasant personal obligations as more inherently enjoyable and raise vitality levels. To test whether a newly devised minimalistic motto-goal intervention can make goal striving more attractive (stronger anticipation of activity related incentives) and energize goal-oriented action (increase vitality) in relation to an unpleasant obligation, two experimental studies were conducted. In Study 1 the motto-goal condition led to stronger anticipation of activity related incentives and vitality compared to a distraction task. The effect on vitality was partially mediated by a change in feelings of autonomy. Study 2 replicated the effects compared to a placebo intervention and further found motto-goals to be specifically effective in increasing the anticipation of activity related incentives as opposed to outcome related incentives. The results support that applying motto-goals built with a newly developed minimalist motto-goal intervention can influence the subjective experience of individuals faced with a previously unpleasant obligation.

## Introduction

Often duties and obligations — like filing a tax return or studying for an unpleasant exam — elicit little positive affect and feelings of vitality. Instead, such tasks are frequently perceived as unpleasant and pursuing them can even be experienced as tormenting and agonizing.

A common and well-studied way to deal with such tasks is to inhibit or suppress the negative emotions and to do the task anyway. This form of self-regulation is often referred to as situational self-control (e.g., Muraven and Baumeister, 2000; Baumeister and Alquist, 2009; Muraven, 2012; Milyavskaya and Inzlicht, 2017). Research has demonstrated its limits and weaknesses (e.g., Muraven and Baumeister, 2000; Friese et al., 2008; Baumeister and Alquist, 2009; Hagger et al., 2010; Milyavskaya and Inzlicht, 2017). For instance, suppressing negative emotions has been shown to lead to more negative emotions on a subsequent task (Hagger et al., 2010). Further, the use of self-control is associated with low levels of subjective energy (Ryan and Deci, 2008; Inzlicht and Schmeichel, 2012) and is as such detrimental for the motivation and subjective well-being of individuals (Ryan and Deci, 2008; Ryan et al., 2008).

In the present work we investigate an alternative way of dealing with such tasks which may be assumed to be associated with higher levels of zest and subjective well-being. We argue that a specific form of goals, so termed motto-goals (Storch and Krause, 2017), should lead to higher levels of vitality when faced with unpleasant tasks. We will provide a new theoretical approach to explain why motto-goals should be effective in making the perception of task pursuit more attractive and inherently enjoyable. In doing so, we borrow from basic social psychological theory, specifically from research on metaphorical language. Two studies are presented to test the derived theoretical assumptions, documenting the benefits of a newly devised motto-goal intervention for the anticipated incentives in goal striving.

### Unpleasant Obligations

We define an unpleasant obligation as a task, which an actor judges as important and whose pursuit is – simultaneously – expected to be unpleasant. There are two major problems associated with such unpleasant obligations.

First, from a motivational perspective, while the judgment of the task as important can be an indicator of *outcome related incentives*, the expected negative affect inherent in unpleasant obligations indicates a lack of *activity related incentives* (Rheinberg, 2008). Outcome related incentives are incentives related to successful completion of the task and the anticipated consequences and are seen as a form of extrinsic and outcome-focused motivation (Rheinberg, 2008; Touré-Tillery and Fishbach, 2014). Activity related incentives consist of anticipated or actual positive emotions (and lack of negative emotions) during task pursuit (Rheinberg, 2008) and are indicators of intrinsic and process-focused motivation (Rheinberg, 2008; Touré-Tillery and Fishbach, 2014). For example, Schultheiss and Brunstein (1999, 2002; see also Job and Brandstätter, 2009) showed that when goal striving is mentally simulated the elicited affective reaction can be used as an indicator of the incentives that goal striving holds for the individual. Accordingly, research showed that pursuing goals which do not hold this kind of incentive (or even elicit negative affect) are associated with lower subjective well-being (Brunstein et al., 1998) and the risk of developing psychosomatic symptoms (Baumann et al., 2005).

Second, research suggests that unpleasant obligations entail a self-control conflict.^1^ The assessment of the task as important on the one side and the negative emotion on the other presents individuals with competing behavioral tendencies namely approaching (due to the importance) and avoiding (due to the negative emotion) (Myrseth and Fishbach, 2009). The consequences are subjective feelings of conflict (e.g., Strack and Deutsch, 2004; Saunders et al., 2017) that evoke further negative affect (see Inzlicht et al., 2015) accompanied by feelings of low energy and subjective vitality (Ryan and Deci, 2008; Milyavskaya and Inzlicht, 2017).

Beyond the negative feelings, an important reason why low levels of subjective vitality are experienced when faced with a self-control task is that people do not feel autonomous or self-driven but controlled and pressured to act (Ryan and Deci, 2008). In the view of self-determination theory, autonomy represents a basic psychological need which entails feeling free and volitional rather than controlled (Deci and Ryan, 2000). Thwarting the need costs energy and fulfillment enhances energy and subjective vitality (Sheldon et al., 1996; Reis et al., 2000; Ryan and Deci, 2008). Subjective vitality – in turn – is associated with being more active and productive as well as showing higher resilience and mental health (see Ryan and Deci, 2008, for an overview). Likewise, numerous studies have documented the positive effects of feelings of autonomy on motivation, mental health and subjective well-being (see Ryan and Deci, 2017, for an overview; Ryan et al., 2008; Weinstein and Hodgins, 2009; Weinstein and Ryan, 2011; Britton et al., 2014).

Considering these potential detrimental effects of pursuing activities that are not intrinsically pleasant or even associated with negative affect and low subjective vitality, the question arises, whether there is a way to make such activities more inherently enjoyable (i.e., raise activity related incentives) and raise subjective vitality levels. Strategies for dealing with tasks that entail a self-control conflict (such as unpleasant obligations) in a less fatiguing way have already been studied in the past under the umbrella term *effortless self-regulation* (e.g., Gillebaart and De Ridder, 2015). Till now, research has mainly studied the trait-like strategies of people with effective self-regulation strategies and the properties of the task (De Ridder et al., 2012; Milyavskaya et al., 2015). For example, effortless self-regulation is facilitated if the long-term goal is automatically activated when individuals are faced with an obstacle in goal-pursuit (see Fujita, 2011, for a review) or if the task is pursued for autonomous (as opposed to controlled) reasons (Milyavskaya et al., 2015). We propose that one way to solve the conflict perceived by individuals in the pursuit of unpleasant obligations would be to increase activity related incentives, thus making goal pursuit more attractive.

One novel way aiming at making task pursuit more attractive and inherently enjoyable are motto-goals.

### Motto-Goals

**FIGURE 1:**
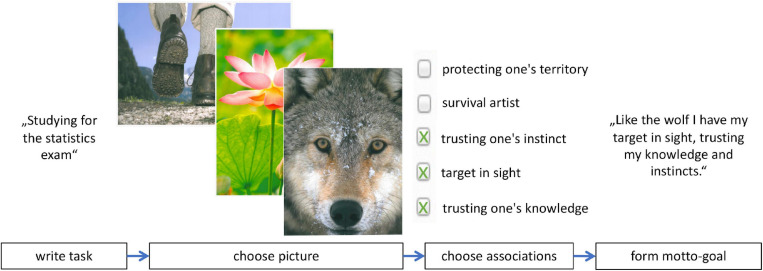
Overview of the motto-goal formation process. Images from Ressourcen aktivieren mit dem Unbewussten: Manual und ZRM-Bildkartei [Activating resources with the unconscious: manual and ZRM picture inventory], by Krause and Storch (2010), Bern: Huber. Images copyright 2010 by Prisma/Dukas. Images reprinted with permission.

Motto-goals describe a desired attitude or mind-set and provide a person with a guiding principle of how to approach a certain topic (e.g., a problem), personal goal or obligation (Storch and Krause, 2017). They are characterized by a specific formation process (Storch and Krause, 2017). In a first step, people choose a subject matter which they would like to handle better (e.g., a task, goal or obligation). In a second step, people are presented with a variety of pictures and are instructed to select a picture which may serve as a resource for the chosen subject matter and which elicits positive affect. In a third step, people are guided to find associations to the chosen picture which elicit positive affect, and in a final step people are instructed to formulate a motto-goal with the chosen associations. For example (see Figure 1), if a person, who had a statistics exam coming up, formed a motto-goal for her unpleasant obligation she could specify “Studying for the statistics exam.” as subject matter. In the second step, she would select a picture which elicits positive affect (e.g., a picture of a wolf). In the third step, she finds associations to the chosen picture which elicit positive affect (e.g., “trusting one’s instinct,” “trusting one’s knowledge,” “target in sight”) and, in the final step, when she is instructed to formulate a motto-goal with the chosen associations she might formulate “Like the wolf I have my target in sight, trusting my knowledge and instincts.” as a desired mind-set.

The term motto-goal was first introduced by Storch (2009). In contrast to goals that describe a desired specific behavior (e.g., high, specific goals; Locke and Latham, 1990), motto-goals describe a desired mind-set and aim to activate a person’s resources (Storch, 2009; Storch and Krause, 2017). They were developed within the framework of the Zurich Resource Model (ZRM; Storch and Krause, 2017) building on dual-process theories (see Strack and Deutsch, 2015, for an overview), Personality Systems Interactions theory (Kuhl, 2000, 2001), Multi Code theory (Bucci, 2002), and the work by Grawe (1998/2004) on resource activation in psychotherapy (for further details see Storch and Krause, 2017). The ZRM is a manualized training, which is typically conducted as a 2-day group training targeting self-management (Storch and Krause, 2017). Research has demonstrated positive effects of the full training on stress and affect regulation abilities in various target groups (Storch et al., 2007, 2011; Storch and Olbrich, 2011; Steurer-Stey et al., 2015).

Although Storch and Krause (2017) see motto-goals as a main active ingredient of the training, until now, only two studies have focused on motto-goals independent of the full training. Weber (2013) examined the effects of a 4-h motto-goal group training. She found that participants in the motto-goal group reported improved affect regulation abilities and higher positive affect compared to two alternative training groups. Rohe et al. (2016) compared motto-goals with learning and performance goals in a complex problem-solving task and found that motto-goals led to higher positive affect and lower negative affect than the other two goal types.

In the present work, we propose that motto-goals have the potential to increase the incentives of goal striving for individuals faced with unpleasant obligations. Our study goes beyond previous studies in numerous ways: First, we will expand previous research on motto-goals by studying them isolated from the group context. This goes beyond the study by Weber (2013) and allows us to disentangle the specific effects of motto-goals from effects of the group context and potential trainer effects (see e.g., Lambert and Barley, 2001). Second, while other research has focused on the trait-like strategies of people with effective self-regulation strategies and the properties of the task (De Ridder et al., 2012; Milyavskaya et al., 2015), we focus on the incentives of goal striving. Third, going beyond the ideas of Rohe et al. (2016) and Storch and Krause (2017), we will expand previous theorizing on motto-goals. Specifically, we propose that motto-goals can be conceptionalized in their core as individually created metaphors with specific characteristics.

To shed light on the process how motto-goals should lead to changes in activity related incentives and vitality levels, we will assimilate theory about motto-goals and metaphors in the next section.

### Metaphors, Motto-Goals and the Characteristics of Their Formation Process

The essence of metaphor is describing “one thing” in terms of “another” (Lakoff and Johnson, 1980). In the property attribution model of metaphor (Glucksberg, 2001), metaphors entail attributing a *property* of a *vehicle* to a *topic*.^2^ For example, in the metaphor “I’m strong as a lion.,” the property “strength” of the vehicle “lion” is attributed to the topic “I.” We propose the formation process of motto-goals can be conceptionalized as creating a metaphor: after selecting a subject matter they would like to handle better, people choose a picture (i.e., a vehicle) and then select associations for the chosen picture (i.e., properties of the vehicle). Finally, they use the associations to formulate their desired mind-set for the chosen subject matter (i.e., they attribute the properties to the topic). In this way, the formation process makes motto-goals metaphors.

To our knowledge, our research on motto-goals is unique in the sense that individually created metaphors are used to alter the emotional and motivational experience of individuals faced with unpleasant obligations. Why should such metaphors help in altering the emotional and motivational experience of individuals? We propose that (1) metaphors and the special way of building motto-goals will make activity related incentives more accessible and that (2) the special quality of the resulting metaphor will enhance vitality and feelings of autonomy in the context of unpleasant obligations.

#### Accessibility of Activity Related Incentives

Lee and Schwarz (2014) proposed that one mechanism of metaphorical language is making metaphor consistent information more accessible and information not consistent with the metaphor less accessible. For example, Hauser and Schwarz (2015) found that “enemy” metaphors in cancer information (e.g., “the war on cancer”) lessened the accessibility of preventive behaviors which are usually not associated with fighting. This view fits with conceptual metaphor theory (Lakoff and Johnson, 1980), which proposes that metaphors highlight certain aspects of metaphorically related concepts and deemphasize other aspects.

Building on this knowledge, we will argue that the process of building motto-goals will ensure that the resulting metaphors will make activity related incentives, such as positive affect, awareness of own resources and personal values more accessible. Central to the process of motto-goal formation is that individuals choose a vehicle (i.e., a picture) and properties (i.e., associations) that elicit positive affect (Storch and Krause, 2017). Storch and Krause (2017) build on work from Grawe (1998/2004) on resource activation in psychotherapy and propose that the focus on positive affect ensures resource activation. While resource activation is seen as an important mechanism of change in psychotherapy (e.g., Gassmann and Grawe, 2006), what entails a resource is often vaguely defined as anything helpful to the individual (e.g., Nestmann, 1996). Building on work from dual process theories of information processing, Storch (2004) proposed that the presence of positive affect could be used as a more objective indicator to resources of an individual because positive affect should be associated with something the person values or past positive experiences of the person. Complementing this view, research on metaphorical framing proposes that positive affect elicited by the vehicle ensures that the metaphor contains something of personal value to the individual (Ottati et al., 1999). While Ottati et al. (1999) studied the effects of a given metaphor, in motto-goal formation people are provided with a variety of possible vehicles and attributes and they choose the vehicle and attributes that elicit positive affect for them. This idiosyncratic approach should ensure that individuals create a metaphor that contains positive personal value associations for them.

Another special feature of the process of formation of a motto-goal is that the resulting metaphor refers to a desired mind-set. According to Crum and Zuckerman (2017) a mind-set can be defined as a lens or frame of mind which orients an individual to a particular set of associations and expectations. According to Storch and Krause (2017) a motto-goal provides a person with a guiding principle of how to approach a task, personal goal or obligation. Importantly, Storch and Krause (2017) do not propose that forming the motto-goal *per se* leads to a change in mind-set by itself, but that the motto-goal has to be mentally applied to the task, personal goal or obligation to exert its effect.

Thus, as metaphors make metaphor-consistent information more accessible (as outlined above) and the process of motto-goal formation focuses on positive affect and they describe a guiding principle of how to approach the obligation, applying a motto-goal to an unpleasant obligation should make activity related incentives more accessible^3^.

#### Vitality and Feelings of Autonomy

Increasing activity related incentives via metaphors should also affect subjective vitality levels of individuals. As outlined before, unpleasant obligations incorporate a self-control conflict (the judgment of the task as “important” on the one hand and negative emotions on the other) and are associated with low subjective energization (Milyavskaya and Inzlicht, 2017). When this conflict is alleviated by increasing activity related incentives (leaving the judgment of “important” on one side and increasing positive emotion on the other), this should be accompanied by higher levels of subjective vitality. Building on research on the factors that influence subjective vitality levels in the context of self-control tasks (Ryan and Deci, 2008), we would expect a change in feelings of autonomy to further contribute to this change in subjective vitality. Because increased activity related incentives make unpleasant obligations more inherently rewarding (i.e., increase intrinsic motivation), the locus of causality should change, and people should feel less controlled in their actions and more autonomous (i.e., the basic need for autonomy should be less thwarted) and this, in turn, should result in stronger feelings of autonomy.

In sum, a motto-goal describes a guiding principle of how to approach an unpleasant obligation. Due to the metaphorical language that explicitly focuses on the elicitation of positive affect, we hypothesize that approaching an obligation with a motto-goal should lead to increased activity related incentives and higher subjective vitality, thus making task pursuit more attractive and inherently enjoyable. We predicted higher feelings of autonomy to contribute to the change in subjective vitality levels^4^.

## The Present Research

The broad aim of our studies was to further understand factors that influence effortless self-regulation while pursuing unpleasant obligations by focusing on an intervention that targets activity related incentives. Furthermore, effects of motto-goals – until now – have hardly been studied apart from potential confounding variables (e.g., group setting). Following our theoretical analyses of motto-goals as individual metaphors with specific characteristics, they should be effective independently of the training, trainer and group situation. We started our research by developing a minimalistic low-cost motto-goal intervention focusing on the core steps necessary to build an individual metaphor while keeping the specific characteristics of motto-goals. To test whether applying motto-goals built within the minimalistic motto-goal intervention increased activity related incentives (anticipated positive incentives of goal striving) and energized behavior regarding an unpleasant obligation.

We also aimed at getting a closer look at the mechanisms driving the effect (1) via mediation analyses and (2) with experimental procedures:

(1) Via mediation analyses, we tested feelings of autonomy as a possible mediator for the effects of motto-goals on subjective vitality (Study 1 and 2).
(2) By varying control groups, we explored whether the effects were due to the specific characteristics and the formation of motto-goals. In Study 1, we compared the motto-goal condition with distraction, a potential coping mechanism when faced with unpleasant emotions. In Study 2, we compared the motto-goal condition with a placebo control group to see whether the effects could be attributed to demand characteristics.

## General Methods

To study the predicted effects of motto-goals in a series of experiments, we needed a minimalistic motto-goal intervention. This intervention was to focus on the core steps theoretically necessary to form an individual metaphor while maintaining all the specific characteristics of the formation process of motto-goals: the use of pictures as vehicles, the focus on positive affect during the choice of the vehicle and the properties, as well as a desired mind-set as a topic.

The final intervention consisted of three phases (see Figure 1 and Supplementary Material for full instructions). Before the intervention, participants were asked to write down an unpleasant obligation which was important to them and which they would like to handle better. Then the intervention began: In the first phase, participants were informed about the procedure used in the intervention. They would be presented with photographs and their task would be to choose one picture that could act as a resource for them. The picture which elicited the highest positive affect would make a suitable resource. Then a set of 10 pictures was presented one after the other in random order. Afterward, participants were to choose a picture based on an overview with thumbnails of all pictures (in random order). The pictures were a selection taken from Krause and Storch (2010) and depict a variety of content, such as flora, fauna, landscapes and humans in pleasant situations. The final set of 10 pictures was selected by experienced trainers of the motto-goal method with the idea to ensure a variety of content (see Krause and Storch, 2010).

In phase 2, participants were presented with their chosen picture and with a list of potential positive associations regarding the chosen picture (the associations were presented in random order). Participants were instructed to choose those associations which elicited positive affect for them. As before with the pictures, these lists of positive associations per picture had been created by experienced trainers to ensure a variety of content and mirror associations typically created by other participants in the training (see Supplementary Material for examples).

In the third phase, participants were instructed to formulate a new attitude or mind-set with their chosen associations that could be applied to their obligation. To assist in the formulation process, they were presented with their chosen picture, the chosen associations and with three possible building blocks to start their motto-goal with. “Please formulate a new approach (attitude or mind-set) to your obligation with your favorite ideas. You can use one of the following sentence beginnings or create your own. I want to be like. I want to feel like. I want to act like.”. They were also presented with some examples of motto-goals and were instructed that motto-goals are often highly person-specific and that they should ensure that their motto-goal would elicit positive affect for them.

After participants finished creating their motto-goal, they were presented on screen with their chosen picture, their created motto-goal and the line: “You have now created your personal motto.”

To sum up, after deciding on an unpleasant obligation, the minimalistic motto-goal intervention consists of choosing a positive picture, selecting positive associations for the picture and then formulating a motto-goal with the chosen associations. See Table 1 for examples of motto-goals built with this minimalistic intervention.

**TABLE 1:** Examples of obligations and motto-goals built by participants.

Study	Obligation	Motto-goal
1	“Studying for the exam”	“I want to feel like a wolf who has his goal in focus and trusts in his knowledge and his instincts.”
	“Writing a paper for a seminar”	“I want to grow from my strong roots into the bright light, with lush green leaves, bursting with energy and then to bear bright red fruits.”
	“Writing job applications”	“I want to act like a bear who takes the time he needs.”
2	“Studying for the course assignments”	“I want to feel like a flower that grows at its own pace.”
	“Get up with the alarm clock”	“I allow myself to flirt calmly and happily with the world.”
	“Studying for exams”	“I want to act like a mountaineer, step by step, at my own pace and well prepared to go my own way.”

*Obligation refers to the chosen unpleasant obligation by participants. All motto-goals were built in Study 1 and 2 by participants using the minimalistic motto-goal intervention.*

## Study 1

In Study 1 we pursued two aims: First, to examine the predicted effects of applying a motto-goal in the context of individually selected unpleasant obligations. More precisely we hypothesized that applying a motto-goal built with the minimalistic intervention increases activity related incentives and subjective vitality. Moreover, we predicted a raise in feelings of autonomy to contribute to the expected raise in subjective vitality levels.

Participants were randomly assigned to either a motto-goal condition or a distraction task. The motto-goal condition consisted of the minimalistic motto-goal intervention plus instructions to apply the motto-goal to the selected obligation. As described above, it is important to apply the motto-goal to a concrete situation, in order to be able to study the effects of the mind-set and the predicted changes in individuals. We chose a distraction task as control group because it controls for the possibility that the effect of the intervention would be merely due to distraction from the motto-goal generation procedure. Moreover, although distraction is known to effectively reduce negative emotions in the moment (Thiruchselvam et al., 2011; Paul et al., 2013), we would not expect a strong effect of distraction on our dependent variables because they all refer specifically to the unpleasant obligation and not to emotions or feelings in the moment.

### Methods

#### Participants

We conducted a formal power analysis to determine the target sample size using G^∗^Power Version 3.1.9.4 (see Faul et al., 2007). The power analysis revealed that a sample size of *N* = 68 would provide sufficient power (1 – β > 0.80) to detect a small-to-medium-sized effect (Cohen’s *f* = 0.175; $\eta_{\text{p}}^{2}$ = 0.030). Since this is the first time the minimalistic motto-goal intervention was used, we had no information on previous effect sizes. We oversampled to account for potential participant attribution and low-quality data during the study. The initial sample consisted of 123 students from a large public university in Germany. Participants received either course credit or monetary reimbursement of €4 (at that time approximately $4.60). The study was advertised as a study about handling unpleasant obligations. We excluded 2 participants who did not chose an unpleasant obligation^5^. This left 121 participants in the final sample (51 female, 70 male; age 17–66, *M* = 23.78, *SD* = 5.55). Sensitivity analyses showed that with the 121 participants we would have sufficient power to detect an effect of Cohen’s *f* = 0.13 ($\eta_{\text{p}}^{2}$ = 0.017).

#### Design

Participants filled out baseline measures (Time 1) and were then randomly assigned to a motto-goal condition (*n* = 61) or to a distraction condition (*n* = 60). After completing the required tasks in each condition, post-measures (Time 2 subjective vitality, activity related incentives, and feelings of autonomy) were assessed. Study 1, therefore, consists of a mixed factorial design with one between factor (Condition: motto-goal vs. distraction) and one within factor (Time: Time 1 vs. Time 2).

#### Procedure

Participants were seated at individual desktop computers in private cubicles. After giving written consent, participants were asked to “… write down an unpleasant obligation which is important for you and which you would like to handle better (e.g., studying for an exam, writing a paper for a seminar).” Then, participants completed baseline measures before being randomly assigned to one of the two experimental conditions:

##### Motto-goal condition

Participants in the motto-goal condition conducted the minimalistic motto-goal intervention as described before. After completing the intervention, participants were asked to write down their motto-goal on a blank sheet of paper.^6^ In order for participants to apply the motto-goal to their chosen obligation, participants received instructions after completing the intervention to imagine themselves working on their obligation with their motto-goal (“Imagine yourself working at fulfilling your obligation with your motto.”).

##### Distraction condition

Participants in the distraction task condition were shown a short historical text about a local city (see Supplementary Material) and were asked to copy the text by hand onto a blank sheet of paper. The condition was pretested to take approximately the same amount of time as the motto-goal condition. After completing the distraction task, participants received instructions to imagine themselves working on their obligation (“Imagine yourself working at fulfilling your obligation.”). This was done to equate conditions.

After completing their respective task, participants filled out post-measures, along with demographics.

#### Measures

At baseline- and post-measurement the chosen obligation was referred to at the beginning of each page (“My obligation: [obligation]”). Additionally, at post-measurement for participants in the motto-goal condition the chosen picture was presented at the beginning of each page and the motto-goal was written under the picture (“My motto: [motto-goal]”).

##### Affective and cognitive attitude toward the obligation

To measure whether participants actually chose an unpleasant and important obligation, we used an affective and cognitive attitude scale based on Brand (2006) measured at Time 1. The original scale measures the affective and cognitive attitude toward doing sports; we adapted it to measure the affective and cognitive attitude toward an obligation by replacing the words “doing sports” with “working on my obligation.” To measure the affective attitude, participants responded on 8-point scales to the statement “When I think of working on my obligation, I feel …”. The endpoints of the scales were: *not relaxed* – *very relaxed*; *not content* – *very content*; *not happy* – *very happy*; *not uncomfortable* – *very uncomfortable* (reverse coded). We averaged the items to yield a total score of affective attitude (Cronbach’s α_t__1_ = 0.83). To measure cognitive attitude, participants responded on 8-point scales to the statement “When I think about it, I think that working on my obligation is …”. The endpoints of the scales were: *not reasonable* – *very reasonable*; *not useless* – *very useless* (reverse coded)*; not profitable* – *very profitable*. We averaged the items to yield a total score of cognitive attitude (Cronbach’s α_t__1_ = 0.68).

##### Subjective vitality

To measure how energized and vital participants felt, we used the 6-item version of the Subjective Vitality State Scale (Ryan and Frederick, 1997; Bostic et al., 2000; Bertrams et al., 2020), the most widely used scale to measure subjective vitality (Ryan and Deci, 2008). We adapted the items to measure subjective vitality while thinking of the obligation (e.g., “At this moment, I feel alive and vital” was changed to “When thinking of my obligation, I feel alive and vital”). Responses were given on a 1 (*no agreement*) to 7 (*full agreement*) scale and averaged to yield a total score (Cronbach’s α_t__1_ = 0.88, α_t__2_ = 0.95).

##### Activity related incentives

As an indicator for activity related incentives, we measured anticipated positive emotions (and lack of negative emotions) during task pursuit (Rheinberg, 2008). We used the 21-item mood-adjective checklist (BEF; Kuhl and Kazén, 2019, unpublished). The scale consists of seven subscales (each with three items): pleasantness, activation, relaxation, helplessness (reverse coded), distress (reverse coded), listlessness (reverse coded), and anger (reverse coded). Participants indicated the intensity of these affective states when imagining working at their obligation (“Imagine working at fulfilling your obligation. How would you feel?”) on a scale from 1 (*very slightly or not at all*) to 5 (*extremely*). We averaged the items to yield a total score (Cronbach’s α_t__1_ = 0.89, α_t__2_ = 0.94).

##### Feelings of autonomy

To measure how autonomous participants felt, we created a 4-item measure consisting of items adapted from autonomy scales from La Guardia et al. (2000), Fröhlich and Kuhl (2003), and Sheldon et al. (2010). The aim was to create a scale measuring feelings of autonomy vs. feelings of being forced when working at fulfilling an obligation. In the final scale, participants read the first part of the statement (“When I work on my obligation, …”) and responded to the items “I feel free.”; “I feel forced.”(reverse coded); “I have to force myself.”(reverse coded); “I can be myself.” on a scale ranging from 1 (*not at all*) to 7 (*extremely*). We averaged the items to yield a total score of feelings of autonomy (Cronbach’s α_t__1_ = 0.65^7^, α_t__2_ = 0.74).

Additional measures collected for exploratory purposes were two open questions about how participants felt when thinking of their obligation (assessed at the beginning of the study directly after writing down their unpleasant obligation), task meaningfulness (assessed after the main measures) and ratings of motivating and soothing effects of the motto-goal (assessed after the main measures). See Supplementary Material for the description of measures and results of the exploratory analyses.

### Results

#### Preliminary Analyses

The reported obligations involved work or university (86%), household (8%) and stress-management (6%). See Table 1 for examples of obligations and motto-goals. At baseline measurement (Time 1), participants reported rather negative affective attitudes (*M* = 3.39, *SD* = 1.32; scale from 1 to 8) and high cognitive attitude scores (*M* = 6.48, *SD* = 1.36; scale from 1 to 8) toward their chosen obligation, reflecting the choice of unpleasant and important obligations. On average participants took 8.25 min (*SD* = 4.04) to complete the motto-goal intervention. See Table 2 for mean values and correlation coefficients of all key variables (see Supplementary Material for mean values separated by experimental condition).

**TABLE 2:** Means, standard deviations, and intercorrelations of all key variables at baseline in study 1.

Variable	*M*	*SD*	1	2
(1) Subjective vitality	2.31	1.07	-	
(2) Activity related incentives	3.23	0.57	0.34***	-
(3) Feelings of autonomy	2.81	1.12	0.32***	0.36***

***p < 0.001.

#### Primary Analyses

##### Subjective vitality

**FIGURE 2:**
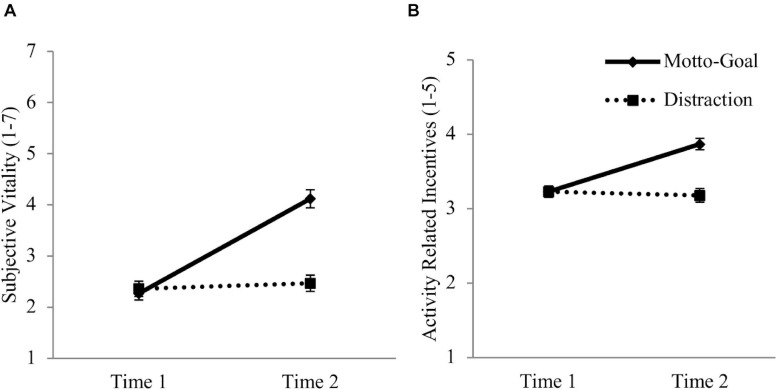
Increase in subjective vitality **(A)** and activity related incentives **(B)** from baseline measurement (Time 1) to post-measurement (Time 2) separated by experimental condition in study 1. Error bars represent standard errors.

We first examined the influence of condition on changes in vitality ratings (see Figure 2A). A mixed GLM with Time (Time 1 vs. Time 2) as a within factor and Condition (motto-goal vs. distraction) as a between factor yielded a significant main effect of Time, *F*(1,119) = 85.50, *p* < 0.001, $\eta_{\text{p}}^{2}$ = 0.418, indicating a significant increase in vitality from Time 1 to Time 2 independent of condition. As expected, a significant interaction effect between Time and Condition emerged, *F*(1,119) = 70.94, *p* < 0.001, $\eta_{\text{p}}^{2}$ = 0.373. This effect indicates that the increase in vitality differed in the conditions. To break down the interaction, paired *t*-tests were performed, comparing the simple main effect for each condition (Field, 2013). In the motto-goal condition vitality ratings increased significantly from Time 1 (*M* = 2.28, *SD* = 1.00) to Time 2 (*M* = 4.28, *SD* = 1.38), *t*(60) = 10.56, *p* < 0.001, *d* = 1.51. In the distraction condition there was no significant change in vitality from Time 1 (*M* = 2.35, *SD* = 1.15) to Time 2 (*M* = 2.44, *SD* = 1.23), *t*(59) = 0.76, *p* = 0.45, *d* = 0.07.

##### Activity related incentives

We tested whether condition influenced changes in activity related incentives (see Figure 2B). A mixed GLM with Time (Time 1 vs. Time 2) as a within factor and Condition (motto-goal vs. distraction) as a between factor yielded a significant main effect of Time, *F*(1,119) = 44.26, *p* < 0.001, $\eta_{\text{p}}^{2}$ = 0.271, indicating a significant increase in activity related incentives from Time 1 to Time 2 independent of condition. As expected, a significant interaction effect between Time and Condition emerged, *F*(1,119) = 59.45, *p* < 0.001, $\eta_{\text{p}}^{2}$ = 0.333. This effect indicates that the increase in activity related incentives differed in the conditions. To break down the interaction, paired *t*-tests were performed, comparing the simple main effect for each condition (Field, 2013). In the motto-goal condition activity related incentives significantly increased from Time 1 (*M* = 3.23, *SD* = 0.57) to Time 2 (*M* = 3.87, *SD* = 0.60), *t*(61) = 8.13, *p* < 0.001, *d* = 1.12. In the distraction condition there was no significant change in activity related incentives from Time 1 (*M* = 3.23, *SD* = 0.58) to Time 2 (*M* = 3.18, *SD* = 0.71), *t*(59) = −1.15, *p* = 0.25, *d* = −0.07.

##### Mediation analyses

**FIGURE 3:**
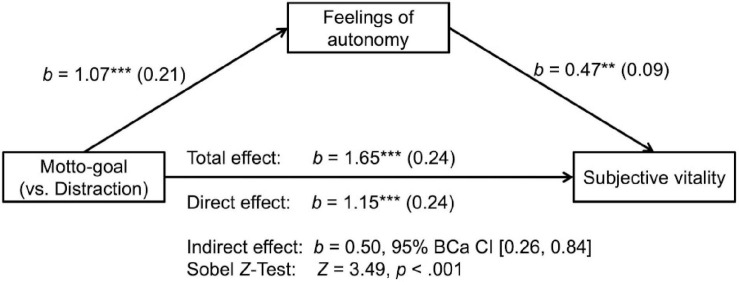
Mediation model for study 1. The predictor variable contrasts the motto-goal condition against the distraction condition (motto-goal 1, distraction 0). Unstandardized coefficients are displayed with standard errors in parentheses. ***p < 0.001, **p < 0.01.

As reported above, the motto-goal condition led to significant increases in ratings of subjective vitality. We performed a mediation analysis to test for an indirect effect of condition on vitality through feelings of autonomy. We conducted this analysis using the PROCESS macro for SPSS (Hayes, 2013) using 10,000 bootstrap samples. Figure 3 illustrates the mediation model and provides path coefficients. As shown, there was a significant indirect effect of condition on vitality through feelings of autonomy, *b* = 0.50, 95% CI [0.26, 0.84].

### Discussion

Study 1 was a first study to investigate the effects of applying a motto-goal to the experience of unpleasant obligations, which was built using a minimalist motto-goal intervention. In line with our assumptions, the results showed an increase in anticipated activity related incentives and in subjective vitality ratings for participants in the motto-goal condition compared to participants in the distraction condition. Further, the increase in subjective vitality was partially mediated by an increase in feelings of autonomy.

We had chosen a distraction condition as a control group because although distraction is seen as a common coping mechanism when faced with unpleasant emotions (e.g., Paul et al., 2013), we did not expect any effects on the dependent variables used in this study. In line with our assumptions, for participants in the distraction condition we found no significant changes in any of our outcome variables (all *p*s > 0.25 and all *d*s < | 0.08|). However, while a distraction task as a control group was a first step in studying the effects of applying a motto-goal, a number of factors could contribute to the effect that are not controlled for by this procedure. For instance, there was no explicit intervention offered to participants in the control group. Thus, the effects of the motto-goal condition could be due to demand characteristics of receiving an intervention. Further, we had made the theoretical assumption that applying a motto-goal would have an effect on activity related incentives rather than on outcome related incentives, because a motto-goal refers specifically to approaching an obligation. While anticipated activity related incentives are an indicator of intrinsic motivation, anticipated outcome related incentives are an indicator of extrinsic motivation (Rheinberg, 2008). Although we found the predicted effect on activity related incentives, including a measure for outcome related incentives would allow to test differential effects on the two kinds of incentives. We designed Study 2 to address these issues.

## Study 2

In Study 2 we pursued three aims: first, to replicate the findings of Study 1, second, to test whether the found effects were stable compared to a placebo intervention that controlled for demand characteristics and third, to examine whether motto-goals are associated with higher anticipated *activity* related incentives rather than higher anticipated *outcome* related incentives.

To pursue the first and the second aim, participants were randomly assigned to a motto-goal condition, a distraction condition or to an expressive writing task as a placebo condition. We chose an expressive writing task as a placebo condition for several reasons. Since there exist several studies showing the effects of expressive writing tasks in unpleasant situations (see Frattaroli, 2006, for a meta-analysis), presenting individuals with an expressive writing task to deal with unpleasant obligations has some validity. Further, expressive writing tasks are considered to be helpful because they allow participants to gain insight into their emotional experience (see Frattaroli, 2006), thus we would not expect a strong effect on our dependent variables, since it is unlikely that gained insights translate into higher levels of subjective vitality and activity related incentives. We would therefore predict that the motto-goal intervention should yield higher scores of activity related incentives and vitality compared to both control groups.

Further, different from Study 1, we did not use a repeated measures design. As preliminary measurement can cause bias (e.g., McCambridge et al., 2011), for example raise awareness of the construct in question and sensitize an intervention. As such demands are addressed in two ways in the present Study: a placebo group and the lack of a preliminary measurement.

To pursue the third aim, we included a measure for anticipated *outcome* related incentives (i.e., incentives related to successful completion of the task and the anticipated consequences). Because a motto-goal applies directly to approaching an obligation, we predicted that applying a motto-goal should specifically influence *activity* related incentives.

### Methods

#### Participants and Design

Given the results in Study 1, we expected at least a medium-sized effect (Cohen’s *f* = 0.25). The power analysis using G^∗^Power Version 3.1.9.4 (see Faul et al., 2007) revealed that a sample size of *N* = 128 would provide sufficient power (1 − β > 0.80) for the planned comparisons. The initial sample consisted of 132 students from a large public university in Germany who either received course credit or monetary reimbursement of €2 (at that time approximately $2.30) for their participation. As in Study 1, the present study was advertised as a study about handling unpleasant obligations. Taking the same approach as in Study 1, we excluded one participant who did not chose an unpleasant obligation. This left 131 participants in the final sample (70 female, 61 male; age 17–45, *M* = 22.25, *SD* = 4.18). Participants filled out initial measures and were then randomly assigned to a motto-goal condition (*n* = 44), to a distraction condition (*n* = 43) or to a placebo condition (*n* = 44). After completing the tasks in the respective conditions, the dependent variables (subjective vitality, activity related incentives, autonomy, and outcome related incentives) were assessed. This represents a factorial design with one between factor (Condition: motto-goal vs. distraction vs. placebo).

### Procedure

Participants arrived in the laboratory and were seated at individual desktop computers in private cubicles. As in Study 1, after giving consent, participants were asked to write down an unpleasant obligation (see Study 1 for detailed instructions). Then, participants completed initial measures before being randomly assigned to one of the three experimental conditions:

#### Motto-Goal Condition

Participants in the motto-goal condition conducted the minimalistic motto-goal intervention as in Study 1. As in Study 1, after completing the intervention participants received instructions to apply the motto-goal to the unpleasant obligation (“Imagine yourself working at fulfilling your obligation with your motto.”).

#### Distraction Condition

Participants in the distraction condition completed the same distraction task as in Study 1. Again, after completing the task participants received instructions to imagine themselves working on their obligation (“Imagine yourself working at fulfilling your obligation.”).

#### Placebo Condition

Participants in the placebo condition conducted an expressive writing task based on Horn et al. (2011). To increase positive expectations of participants and demand characteristics, we included a statement that the task had been shown to be effective by previous research. The initial instructions were: “Next, we would like you to write about your emotions and thoughts about the obligation. Research has shown that this can be very helpful when dealing with unpleasant obligations.” Then the standard instructions of expressive writing followed (see Horn et al., 2011), adapted to writing about an obligation.

In your writing really let go and explore your feelings and thoughts. You might link the obligation “[obligation]” to your relationships with other people, e.g., your partner, your friends or your relatives. Ask yourself how this experience relates to your past, your momentary situation, or your future or who you have been in the past and who you would like to become in the future.

Don’t worry about spelling or grammar. Try to write without interruption until the time is up. Everything you write will be kept strictly confidential.

Following the task participants received instructions to imagine themselves working on their obligation (“Imagine yourself working at fulfilling your obligation.”).

After completing their condition, participants filled out post-measures, along with demographics.

### Measures

At initial measurement and post-measurement, the chosen obligation was referred to at the beginning of each page. Additionally, at post-measurement for participants in the motto-goal condition the motto-goal was displayed.^8^ The initial measurement consisted of the affective attitude (Cronbach’s α = 0.76) and cognitive attitude (Cronbach’s α = 0.78) toward the obligation scales as described in Study 1. The dependent measures of subjective vitality (Cronbach’s α = 0.78), activity related incentives (Cronbach’s α = 0.92), and feelings of autonomy (Cronbach’s α = 0.79) also paralleled those used in Study 1. Additionally, the following measure was assessed:

#### Outcome Related Incentives

As an indicator for outcome related incentives, we measured anticipated positive affect (and lack of negative affect) after task completion (Rheinberg, 2008). We used the 17-item anticipatory affect scale (Bagozzi et al., 1998). The original scale measures the anticipated affect after completing a goal; we adapted it to measure the anticipated affect after completing an obligation by replacing “achieving a goal” with “fulfilling an obligation.” The scale consists of 7 items measuring positive affect after successful goal completion (e.g., “happy,” “glad,” “satisfied”) and 10 items measuring negative affect after goal completion had failed (e.g., “angry,” “sad,” “worried”). The instructions were: “If I succeed at fulfilling my obligation, I will feel…” for the positive items and “If I don’t succeed at fulfilling my obligation, I will feel…” for the negative items. Participants indicated the intensity of their affective state from 1 (*very slightly or not at all*) to 5 (*extremely*). We recoded the negative items and then averaged the items to yield a total score of outcome related incentives (Cronbach’s α = 0.83). High scores indicate high anticipated positive incentives and low negative incentives.

We assessed further variables for exploratory purposes. The variables comprise the same open questions about how participants felt when thinking of their obligation (assessed at the beginning of the study directly after writing down their unpleasant obligation) used in Study 1 and a measure of self-efficacy (assessed after the main variables). See Supplementary Material for the description of measures and results of the exploratory analysis.

### Results

#### Preliminary Analyses

The reported obligations involved work or university (79%), household (8%) and other areas (13%). See Table 1 for examples of obligations and motto-goals. At initial measurement, participants reported rather negative affective attitude scores (*M* = 2.87, *SD* = 1.06; scale from 1 to 8) and high cognitive attitude scores (*M* = 6.77, *SD* = 1.36; scale from 1 to 8) toward their chosen obligation, reflecting unpleasant and important tasks. On average participants took 7.57 min (*SD* = 3.01) to complete the motto-goal intervention. See Table 3 for mean values and correlation coefficients of all key variables (see Supplementary Material for mean values separated by experimental condition).

**TABLE 3:** Means, standard deviations, and intercorrelations of all key variables in study 2.

Variable	*M*	*SD*	1	2	3
(1) Subjective vitality	2.89	1.29	–		
(2) Activity related incentives	3.37	0.64	0.49***	–	
(3) Feelings of autonomy	3.00	1.32	0.51***	0.63***	–
(4) Outcome related incentives	3.52	0.57	0.25**	0.35***	0.38***

***p < 0.001, **p < 0.01.

#### Primary Analyses

We conducted one-way ANOVAs to analyze the effect of condition on the dependent variables. Planned contrasts were used to test the hypotheses. The first contrast compares the motto-goal condition to the control condition (coded as motto-goal = 1, placebo = 0, distraction = −1), and the second contrast compares the motto-goal condition to the placebo condition (coded as motto-goal = 1, placebo = −1, distraction = 0). *Post hoc* comparisons comparing the placebo and the distraction condition are calculated using Dunnet-*T*.

##### Subjective vitality

**FIGURE 4:**
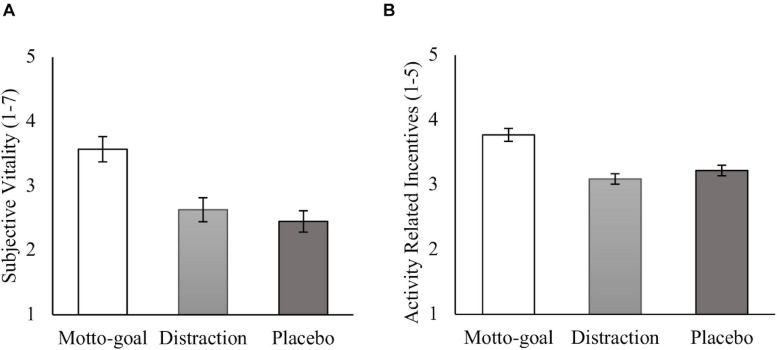
Ratings of subjective vitality **(A)** and activity related incentives **(B)** separated by experimental condition in study 2. Error bars represent standard errors.

We first examined the influence of condition on subjective vitality ratings (see Figure 4A). A one-way ANOVA showed significant differences in subjective vitality ratings across conditions, *F*(2,128) = 10.62, *p* < 0.001, $\eta_{\text{p}}^{2}$ = 0.142. Turning to our central analysis, the planned contrasts described above revealed that participants in the motto-goal condition (*M* = 3.57, *SD* = 1.30) reported significantly higher vitality scores compared to participants in the distraction condition (*M* = 2.66, *SD* = 1.22), *t*(128) = 3.60, *p* < 0.001, *d* = 0.74, and compared to participants in the placebo condition (*M* = 2.45, *SD* = 1.10), *t*(129) = 4.34, *p* < 0.001, *d* = 0.93. *Post hoc* analyses using Dunnett-*T* revealed no significant difference between the placebo condition and the distraction condition (*p* = 0.64).

##### Activity related incentives

We next tested whether condition influenced activity related incentives (see Figure 4B). A one-way ANOVA yielded a significant pattern of differences across conditions, *F*(2,128) = 16.68, *p* < 0.001, $\eta_{\text{p}}^{2}$ = 0.202. Using the planned contrasts described above revealed that the activity related incentives ratings where significantly higher in the motto-goal condition (*M* = 3.77, *SD* = 0.66) compared to the distraction condition (*M* = 3.10, *SD* = 0.53), *t*(128) = 5.45, *p* < 0.001, *d* = 1.17, and compared to the placebo condition (*M* = 3.23, *SD* = 0.54), *t*(128) = 4.38, *p* < 0.001, *d* = 0.93. *Post hoc* analyses using Dunnett-*T* revealed no significant difference between the placebo condition and the distraction condition (*p* = 0.52).

##### Mediation analysis

**FIGURE 5:**
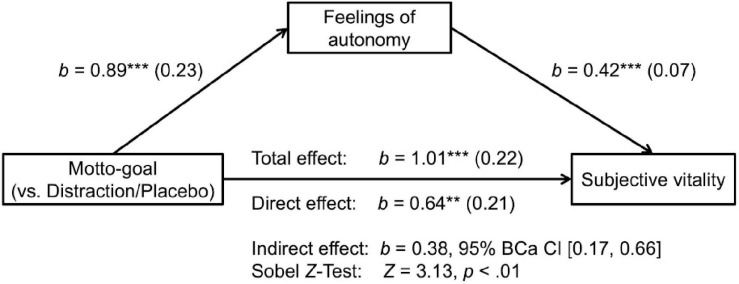
Mediation model for study 2. The predictor variable contrasts the motto-goal condition against the distraction condition and the placebo condition (motto-goal 2, distraction 1, and placebo 1). Unstandardized coefficients are displayed with standard errors in parentheses. ***p < 0.001, **p < 0.01.

As reported above, the motto-goal condition led to significant increases in ratings of subjective vitality. We performed a mediation analysis to test for an indirect effect of condition on vitality through feelings of autonomy using the PROCESS macro for SPSS (Hayes, 2013) using 10,000 bootstrap samples. Figure 5 illustrates the mediation model and provides path coefficients. As shown, there was a significant indirect effect of condition on vitality through feelings of autonomy, *b* = 0.38, 95% CI [0.17, 0.66].

##### Activity related incentives vs. outcome related incentives

**FIGURE 6:**
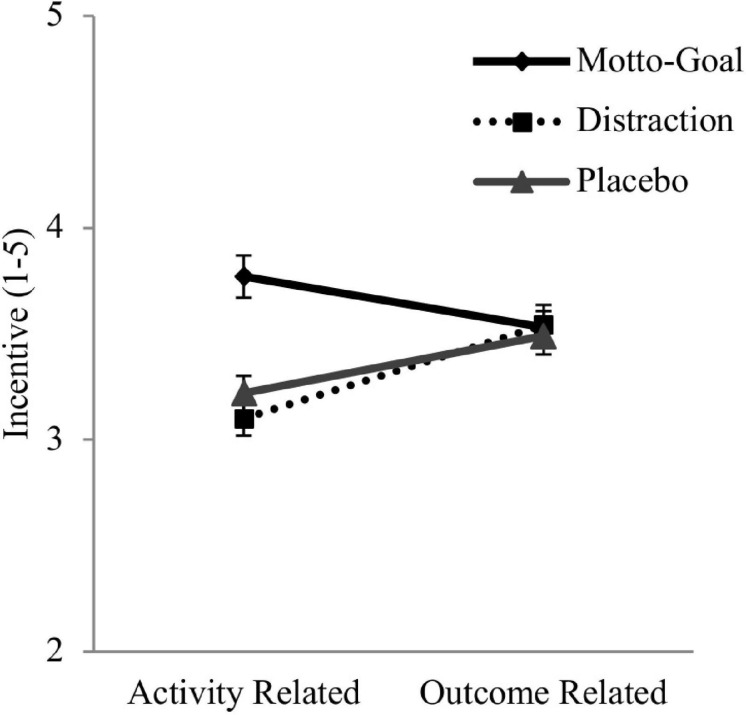
Difference between activity related (activity related) and outcome related (outcome related) incentives separated by experimental condition in study 2. Error bars represent standard errors.

To test whether the motto-goal conditions had effects rather on activity related incentives than outcome related incentives, we tested whether the difference of activity related incentives and outcome related incentives differed between conditions (see Figure 6). We conducted a mixed GLM with Incentive (activity vs. outcome) as a within factor and Condition (motto-goal vs. placebo vs. distraction) as a between factor. There was a significant main effect of Incentive, *F*(1,128) = 7.62, *p* = 0.007, $\eta_{\text{p}}^{2}$ = 0.056. In line with the nature of unpleasant obligations, and independent of condition, activity related incentives were lower than outcome related incentives. Further, a significant interaction effect between type of incentive and condition emerged, *F*(2,128) = 13.84, *p* < 0.001, $\eta_{\text{p}}^{2}$ = 0.167, indicating that, as expected, the balance of activity related incentives and outcome related incentives differed between conditions. To break down the interaction, we performed one-way ANOVAS and compared the simple main effect of Incentive for each condition. As reported above, for activity related incentives a one-way ANOVA showed that in the motto-goal condition participants reported significantly higher levels compared to the other conditions. For outcome related incentive ratings a one-way ANOVA showed no significant differences across conditions, *F*(2,128) = 0.09, *p* = 0.91. Paired *t*-tests to test the simple main effect of Incentive for each condition showed that only in the motto-goal condition there were significantly higher levels of activity related incentives (*M* = 3.77, *SD* = 0.66) than outcome related incentives (*M* = 3.53, *SD* = 0.52), *t*(43) = 2.64, *p* = 0.011, *d* = 0.39. In the distraction condition and in the placebo condition the effect was reversed: There were significantly lower levels of activity related incentives (*M*_*dist*_ = 3.10, *SD_*dist*_* = 0.53; *M*_*plac*_ = 3.23, *SD*_*plac*_ = 0.54) than outcome related incentives (*M*_*dist*_ = 3.54, *SD_*dist*_* = 0.63; *M*_*plac*_ = 3.49, *SD*_*plac*_ = 0.58) [distraction condition: *t*(42) = −4.27, *p* < 0.001, *d* = −0.74; placebo condition: *t*(43) = −2.63, *p* = 0.012, *d* = −0.47].

### Discussion

Study 2 replicated the effects found in Study 1. As expected, levels of subjective vitality and anticipated activity related incentives were higher in the motto-goal condition compared to a distraction condition and also compared to a placebo condition. Moreover, we replicated the indirect effect of the intervention via feelings of autonomy on subjective vitality ratings. Showing that the effects are stable compared to a placebo condition supports the notion that the effects of the motto-goal condition are not merely due to general demand characteristics or general positive expectancies of receiving an intervention (see Haaga and Stiles, 2000), but more specific to the nature of motto-goal application.

Going beyond Study 1, the results showed that the motto-goal condition had effects rather on activity related incentives than outcome related incentives and that the balance of activity related to outcome related incentives differed between conditions. Only in the motto-goal condition were the activity related incentives higher than the outcome related incentives. This indicates that in the motto-goal condition the incentives deriving from the task (intrinsic motivation) were higher than the positive incentives from task completion (extrinsic motivation) (Rheinberg, 2008; Touré-Tillery and Fishbach, 2014). Even though it is questionable whether motto-goals would always lead to higher activity related incentives than outcome related incentives, the results illustrate that motto-goals change the balance of type of incentive toward higher activity related incentives, indicating positive aspects of approaching the obligation being more accessible.

## General Discussion

Even when goals are important for us, goal striving can be unpleasant and can even be experienced as tormenting. Research suggests that this lack of activity related incentives, low levels of subjective energy and feelings of autonomy can be detrimental for our mental health and subjective well-being (Brunstein et al., 1998; Ryan and Bernstein, 2004; Baumann et al., 2005; Weinstein and Ryan, 2011; Desteno et al., 2013).

In the current investigation, we examined for the first time whether applying a motto-goal built with a newly devised motto-goal intervention can change the perceived incentives inherent in goal striving — and as such enrich the literature on effortless self-regulation strategies (Gillebaart and De Ridder, 2015). Given that the formation process of motto-goals can be conceptionalized as creating an individual metaphor with focus on positive affect during the whole process, pictures as vehicles and a desired mind-set as the target, we reasoned that applying a motto-goal should lead to perceiving an obligation with more activity related incentives and feelings of vitality. The results of two randomized controlled laboratory studies lend support to these central hypotheses. These findings highlight that motto-goals can influence the subjective experience of individuals faced with a previously unpleasant obligation, making goal striving seem more attractive and energizing. Across our studies, we found that the effects of the motto-goal condition on subjective vitality are partially explained by feelings of autonomy (Study 1 and 2). It would seem, as hypothesized, that motto-goals lead to more subjective vitality by changing the focus to more intrinsically rewarding aspects of pursuing an obligation and thus increasing autonomous motivation (Ryan and Deci, 2008). In line with this idea, we found motto-goals to be specifically effective on increasing anticipated activity related incentives (an indicator of intrinsic motivation) as opposed to outcome related incentives (an indicator of extrinsic motivation) (Study 2). An important advantage of the present studies is the varying control groups to exclude other potential explanations. We documented the results compared to a distraction task, a potential coping mechanism when faced with unpleasant emotions (Study 1) and a placebo control group, to rule out demand characteristics (Study 2).

### Implications

Past theory and research argue that facing unpleasant obligations, defined as tasks which are judged as important and are experienced as unpleasant at the same time, by inhibiting or suppressing negative emotions (i.e., applying self-control) can have detrimental effects on our motivation and subjective well-being (Ryan and Deci, 2008; Hagger et al., 2010; Inzlicht and Schmeichel, 2012; Milyavskaya and Inzlicht, 2017). Our findings show that motto-goals can change the subjective experience of individuals faced with such unpleasant obligations: Individuals report being more energetic and vital when thinking of the obligation (Study 1 and 2) and report higher anticipated activity related incentives (Study 1 and 2). Our findings contribute to the growing field of research interested in more effortless self-regulation strategies (for a review Gillebaart and De Ridder, 2015). For example, research has studied the creation of habits to establish automatic routines (Galla and Duckworth, 2015) and the creation of automatic associations to the long-term goal, which are activated when individuals face an obstacle in goal-pursuit (see Fujita, 2011, for a review). Going beyond previous research which focused on the characteristics of the long-term goal (Milyavskaya et al., 2015), we focus on the incentives in goal striving. Showing that the way to the goal is perceived differently with a motto-goal. Specifically, our results fit with research documenting that people with effective and relatively effortless self-regulation strategies on a trait level often solve self-control conflicts by making goal pursuit more attractive (see Gillebaart and De Ridder, 2015). Our results expand this previous research by demonstrating motto-goals as a tool where this can be accomplished as an intervention.

Further, to our knowledge, the use of metaphorical language has not yet been studied in self-regulation research as a means to solve self-control conflicts. Our findings and theoretical analyses on metaphorical framing point in exciting possible new directions: even independently of motto-goals research, we suggest metaphorical language should be studied more as a possible means to solve self-control conflicts.

In addition, our results may show a possible way to more healthy goal striving. Apart from the motivational implications of making goal pursuit more attractive (i.e., increasing intrinsic motivation), the experience of positive affect has beneficial effects on the health of individuals (Lyubomirsky et al., 2005; Desteno et al., 2013). For example, the daily experience of positive affect predicts increases in trait resilience, which is associated with improved subjective well-being (Cohn et al., 2009). Moreover, subjective vitality is not only an indicator of motivation (Ryan and Frederick, 1997; Ryan and Deci, 2008) but also of mental health and optimal functioning (see Ryan and Bernstein, 2004, for an overview). Further, increased feelings of autonomy are not only our predicted mechanism of change in vitality levels but also have implications for mental health (see Ryan and Deci, 2017, for an overview). For example, feelings of autonomy are associated with the effective processing of stressful events (Weinstein and Hodgins, 2009). Our findings contribute to research in self-determination theory (Deci and Ryan, 2000) on what influences subjective vitality levels and feelings of autonomy. Our results suggest that it can be a changed mind-set of the individual on the task at hand. To sum up, motto-goals seem to promote a healthier focus on goal striving.

The present research also contributes to the literature on motto-goals and metaphors. Motto-goals are usually applied as part of a 2-day group training targeting self-management (Storch and Krause, 2017). Research on the group training indicates positive effects, for example, on stress (e.g., Storch et al., 2007). Our results expand previous research in several ways: First, we expanded the theoretical foundation of motto-goals. We theoretically analyzed motto-goals from the perspective of research on metaphorical framing (e.g., Landau et al., 2014) and concluded that motto-goals can be conceptionalized as personal metaphors, which are formed in a specific way: with a picture as a vehicle, a focus on positive affect during the whole formation process, and as topic a desired mind-set of the individual (see Glucksberg, 2001). Our analysis offers insights into the psychological processes involved in the effects of motto-goals that can help guide future studies (this will be discussed further in the “Limitations and Future Research” section). Second, we developed a minimalistic motto-goal intervention, in which individuals can form a motto-goal at a computer. The minimalistic intervention focusses on the core steps necessary to form an individual metaphor while maintaining the specific characteristics of the formation process of motto-goals. This is the first time that individually created metaphors — built by a standardized formation process — are used as an intervention in self-regulation research.

### Limitations and Future Directions

From the viewpoint of metaphor research, more precisely the research on linguistically framing concepts with metaphorical language, we identified one key mechanism of the effects of metaphorical language: changing the accessibility of information by making metaphor consistent information more accessible (e.g., Lee and Schwarz, 2014). In the present study, we laid the theoretical groundwork for future studies and took first steps by finding that motto-goals make positive aspects of approaching an obligation more accessible (Study 1 and Study 2). Extending our theoretical analyses, researchers have further proposed that metaphoric language (vs. literal language) elicits vivid and evocative mental images (Paivio, 1971; Ortony, 1975; Billow, 1977; Graesser et al., 1989; Gibbs, 2014). This could make metaphors especially effective in eliciting emotions and feelings (see Ji et al., 2016, for a review). In future studies it could be explored if motto-goals elicit their effects through a change of mental images.

From a motivational perspective, a further key mechanism could be the activation of implicit needs of participants. Storch and Krause (2017) assume that the positive affect that participants experience when choosing a picture, and later positive associations, is an indicator of activated implicit needs of participants. In the full training participants are guided to reflect upon what need is expressed for them in the chosen picture and in the chosen associations (Storch and Krause, 2017). There is support for the idea that affect can represent implicit needs of individuals (e.g., Schultheiss, 2001; see also Job and Brandstätter, 2009) and that the exploration of the affective reaction, for example toward a picture, can lead to insights about the needs of the person (e.g., Bucci, 2002; Schultheiss and Strasser, 2012). In future research, it could be explored whether the affective reaction of participants when forming the motto-goal is connected to their implicit needs and whether the additional step of reflecting upon what need is expressed influences the effectiveness.

While we found promising results for motto-goals built with the minimalistic intervention in the context of unpleasant obligations, future studies should also examine the effects in new contexts. For example, staying close to self-regulation research, it could be studied whether motto-goals can also help individuals in a “temptation scenario” (Hofmann et al., 2011). While unpleasant obligations entail overcoming a negative impulse (e.g., the aversiveness of studying for an exam) to pursue a long-term goal (e.g., graduation), a “temptation scenario” entails resisting an attractive impulse (e.g., eating a cookie) to pursue a long-term goal (e.g., losing weight). Our predictions would be that motto-goals could be applied to bring positive affect and feelings of autonomy to pursuing the long-term goal (as we showed for unpleasant obligations in the present studies) and thus should also be effective in this context. In support of our idea, research shows that making goal pursuit more attractive (e.g., by more autonomous motivation) reduces temptations before they are experienced and thus obviates the need for self-control (Milyavskaya et al., 2015).

Since we were interested in the question whether motto-goals can change the subjective experience of individuals, we used self-report measures. While there are of course some limitations to self-report measures (e.g., Wilson and Brekke, 1994) for our goal of measuring the subjective experience of individuals they seemed the method of choice and in accordance with this view Lisa Feldman Barrett states that “self-report represents the most reliable and possibly only window that researchers have on conscious, subjective, emotional experience” (Barrett, 1996, p. 47). Nevertheless, future research could extend our results and use implicit measures to measure core affect (see Russell, 2003) and automatic associations (see Nosek et al., 2011, for a review of implicit measures and the processes underlying them). This would give the possibility to see whether also automatic associations and evaluations of individuals are influenced.

In our studies individuals were faced with unpleasant obligations *in sensu* (imagining goal striving) and not *in vivo* (actual goal striving). Participants were asked to think about pending obligations that were unpleasant and important to them. As such, our studies show that motto-goals lead to changed expectations and associations regarding personally relevant unpleasant obligations, namely the anticipation of activity related incentives and vitality. While anticipated affective reactions have been shown to be valid indicators of motivation (e.g., Bagozzi et al., 1998; Schultheiss and Brunstein, 1999) and mentally simulating goal pursuit can evoke similar psychological consequences as the corresponding experience in reality (e.g., Kappes and Morewedge, 2016), research on affective forecasting has shown that individuals usually show some bias in the prospection of future affective states. For example, Gilbert and Wilson (2007) summarize research showing that individuals often overestimate the intensity of affective states compared to the actual experience. Future research should study if and how the subjective experience during goal striving (e.g., experienced activity related incentives) is changed by applying motto-goals. Building on research on affective forecasting we would expect the same direction of effects but with smaller effect sizes than in the presented studies.

According to Storch and Krause (2017) motto-goals act as a resource for individuals, which they can apply when in need. The mind-set that motto-goals describe should change the perception and expectations of individuals. Our studies provide first evidence that motto-goals lead to the perception of more activity related incentives and vitality in the context of unpleasant obligations. While this is an important first step, future studies should study if the minimalistic motto-goal intervention can be successful as an intervention in the field, changing the subjective experience of individuals and guiding behavior. In this context it is important to point out, that forming a motto-goal is not proposed to lead to a lasting change in mind-set *per se*, but that the motto-goal has to be made temporarily accessible to exert its effect (Storch and Krause, 2017). This approach is in line with research on metaphorical framing, which also suggests that the metaphor has to be available in the situation to have an effect (e.g., Hauser and Schwarz, 2015). Therefore, researchers interested in conducting field-experiments and testing long-term effects should take measures for making the motto-goal temporarily accessible for individuals (see Storch and Krause, 2017, for suggestions on how this can be accomplished).

### Conclusion

Motto-goals are personal metaphors that describe a desired mind-set and provide a person with a guiding principle of how to approach a certain topic, personal goal or obligation. Our investigation indicates that applying motto-goals built with a newly developed minimalist motto-goal intervention can change people’s subjective experience when faced with an unpleasant obligation. Future research should build on these findings to further uncover the ways in which motto-goals shift people away from a negative subjective experience, toward a focus on more positive aspects of goal striving.

## Data Availability Statement

The raw data supporting the conclusions of this article will be made available by the authors, without undue reservation.

## Ethics Statement

Ethical review and approval was not required for the study on human participants in accordance with the local legislation and institutional requirements. The patients/participants provided their written informed consent to participate in this study.

## Author Contributions

TD developed the study concept and design and collected the data. OD supervised the study. TD, OD, and DS analyzed and interpreted the data. TD drafted the manuscript. DS and OD critically revised the manuscript. All authors approved the final version to be published and agreed to be accountable for all aspects of the work in ensuring that questions related to the accuracy or integrity of any part of the work are appropriately investigated and resolved.

## Conflict of Interest

The authors declare that the research was conducted in the absence of any commercial or financial relationships that could be construed as a potential conflict of interest.

## Footnotes

1 As opposed to a “temptation scenario,” which entails resisting an attractive impulse (e.g., eating a cookie) to pursue a long-term goal (e.g., losing weight), this scenario entails overcoming a negative impulse (e.g., the aversion to studying for the exam) to pursue a long-term goal (e.g., graduation) and has been termed “heroism scenario” (Hofmann et al., 2011).

2 In conceptual metaphor theory (Lakoff and Johnson, 1980) the labels *source* and *target* are used instead of *vehicle* and *topic*.

3 In the context of unpleasant obligations (tasks with high outcome related incentives and low activity related incentives) we would not expect a strong effect on outcome related incentives since they are already accessible for individuals.

4 For a mediation between autonomy and activity related incentives, theories were not as straightforward. From a theoretical standpoint it could be argued that the change in activity related incentives leads to a change in autonomy and/or that a change in autonomy leads to activity-related incentives.

5 To lose as few participants as possible, we took a conservative approach and decided to exclude only participants with an affective attitude score toward their obligation at baseline measurement of between 7 and 8 (on a scale from 1 to 8, 8 representing a pleasant obligation). Since we explicitly advised participants to choose an unpleasant obligation, this is also likely to reflect inattentive completion of the study.

6 This was done to equate conditions and not to raise suspicion since the sheet of paper was used in the distraction condition.

7 All items contributed to the reliability (see Supplementary Material).

8 In Study 2, we only displayed the motto-goal and not the chosen picture.
