# Supplementary material for: Personal Metaphors as Motivational Resources: Boosting Anticipated Incentives and Feelings of Vitality Through a Personal Motto-Goal
Source: Front Psychol. 2021 Apr 13;12:566215. doi: 10.3389/fpsyg.2021.566215 (PMC8076612; doi:10.3389/fpsyg.2021.566215)
Supplement: Supplementary file 1 [file Data_Sheet_1.docx]

Supplementary Material

# Means and Standard Deviations of All Key Variables Separated by Experimental Condition

|  |  | Study 1 (*N* = 121) | | | | |  | Study 2 (*N* = 131) | | |  |
| --- | --- | --- | --- | --- | --- | --- | --- | --- | --- | --- | --- |
|  |  | Time 1 | |  | Time 2 | |  |  |  |  |  |
| Variable |  | Motto-Goal | Distrac-tion |  | Motto-Goal | Distrac-tion |  | Motto-Goal | Placebo | Distrac-tion |  |
|  |  | *M*  *(SD)* | *M*  *(SD)* |  | *M*  *(SD)* | *M*  *(SD)* |  | *M*  *(SD)* | *M*  *(SD)* | *M*  *(SD)* |  |
| Subjective Vitality |  | 2.28 (1.00) | 2.35 (1.15) |  | 4.28 (1.38) | 2.44 (1.23) |  | 3.57 (1.30) | 2.45 (1.10) | 2.66 (1.22) |  |
| Activity Related Incentives |  | 3.23 (0.57) | 3.23 (0.58) |  | 3.87 (0.60) | 3.18 (0.71) |  | 3.77 (0.66) | 3.23 (0.54) | 3.10 (0.53) |  |
| Feelings of Autonomy |  | 2.83 (1.25) | 2.78 (0.98) |  | 3.93 (1.29) | 2.85 (1.00) |  | 3.59 (1.47) | 2.45 (1.04) | 2.95 (1.17) |  |
| Outcome Related Incentives |  |  |  |  |  |  |  | 3.53 (0.52) | 3.49 (0.58) | 3.54 (0.63) |  |

# Instructions of the Minimalistic Motto-Goal Intervention

| German (original) | English (translation) |
| --- | --- |
| In einem ersten Schritt wirst du verschiedene Bilder sehen, von welchen du eines auswählen kannst.  Bitte wähle nach folgendem Kriterium: Welches Bild kann dir als Ressource mit „[gewählte Aufgabe]“ dienen?  Wähle "dein" Bild schnell und spontan. Ein Bild ist geeignet, wenn es in dir spontan ein starkes gutes Gefühl auslöst (z.B. Freude, wirkt beruhigend, lässt dich lächeln, ...).  Bitte achte darauf, dass du nach „deinem Bauchgefühl“ auswählst, selbst wenn du rational noch nicht ganz nachvollziehen kannst, was das Bild und das gute Gefühl mit „[gewählte Aufgabe]“ zu tun haben. | In a first step you are about to see various pictures, from which you may select one.  Please select according to the following criterion: Which picture can serve you as resource with "[chosen task]" ?  Please select "your" picture based on your feeling, quickly and spontaneously. A picture is suitable as resource if it spontaneously gives you a strong positive feeling (e.g. joy, feels calming, makes you smile, …).  Please make sure you select according to "your gut feeling", even if you do not completely rationally understand what the picture and the positive feeling have to do with "[chosen task]". |
| Bitte klicken Sie nun auf das Bild, das bei Ihnen ein positives Gefühl auslöst und dann auf "weiter".  Falls mehrere Bilder ein gutes Gefühl auslösen, dann wählen Sie bitte das Bild, welches das stärkste gute Gefühl auslöst. | Please now click on the picture that gives you a positive feeling and then on "next".  In case several pictures make you feel good, then please select the picture that gives you the strongest good feeling. |
| Meine gewünschte innere Haltung  Bitte formulieren Sie mit Ihren Lieblingsideen eine gewünschte innere Haltung. Dabei soll es nicht um konkrete Verhaltensweisen gehen, sondern um eine innere Haltung, also wie Sie sich fühlen, handeln oder sein wollen.  Beispiele für eine gewünschte innerer Haltung:  *Ich will mich fühlen wie ein Bär, der ein dickes Fell hat.* *Ich will handeln wie die junge Frau auf der Vespa, frech und Vollgas voraus.*  *Ich will sein wie der Lotus und in mir fest verwurzelt im Licht wachsen, um in meine volle Blüte zu kommen.*  Die Sätze unterscheiden sich in Länge und "Blumigkeit" je nach individuellem Typ. Achten Sie darauf, dass ihre Version bei Ihnen ein gutes Gefühl auslöst.  Sie können dabei einen der folgenden Satzbausteine benutzen oder einen eigenen Satz bilden.  Ich will mich fühlen wie…  Ich will handeln wie…  Ich will sein wie… | My desired mindset  Using your favourite associations please formulate a desired mindset. Here, do not focus on a specific behaviour but a certain attitude to life.  Examples for a desired mindset: *I want to feel like a bear that has a thick coat.  I want to act like the young woman on the scooter, bold and full speed ahead. I want to be like a lotus and, firmly rooted in myself, grow in the light in order to come into full bloom.*  The sentences vary in length and "floweriness" depending on personal preference. Take care that your version makes you feel good.  You can begin the sentence with one of the following phrases or you can use your own words.   I want to feel like… I want to act like… I want to be like… |

# Examples of Lists of Associations

|  | German (original) | English (translation) |
| --- | --- | --- |
| Picture of a wolf | - fokussiert - instinktsicher - das Ziel im Visier, - findet sich im Rudel zurecht - Überlebenskünstler - freiheitsliebend - Beute aufspüren - schlau - Spur verfolgen - guter Jäger - Urinstinkte - Wolfsinstinkt - vertraut auf sein Wissen - schnappt sich seine Beute - hört auf seine Bedürfnisse - Langstreckenläufer und guter Sprinter - geht seinen Weg - Einzelgänger - nimmt sich was er braucht - auf leisen Pfoten - wacher Blick - schützendes Fell - klarer Blick, gutes Gehör - findet Geborgenheit im Wolfsbau - robust - wandert grosse Strecken - guter Orientierungssinn - schützt sein Revier - treuer Begleiter und Beschützer | - focused - trusting one’s instinct - target in sight - knowing one's place in   the pack   - survival artist - freedom loving - tracking prey - cunning - smart - following the track - good hunter - primal instinct - wolf instinct - trusting one's knowledge - snatching the prey - listens to his needs - long-distance runner and good sprinter - going his way - lone wolf - loner - takes what he needs - on soft paws - alert mind - protective coat - acute vision - good hearing - feeling safe in the wolf den - robust - travelling great distances - good sense of orientation - protecting one's territory - faithful companion and guardian |
| Picture of a flower | - Strahlen - im Zentrum stehen - ich zeige meine Pracht - aufblühen - im Licht wachsen - stark und zart zugleich - die Schönste weit und breit - neue Triebe - Blatt als Schutz für die Knospe - duftet süss - lustvolles Öffnen zur Sonne - fest verwurzelt auf der Oberfläche schwimmen - lotusfreundliche Gewässer - Wachstum - Transparenz - in Mitten von Vielen - erstrahlt in voller Blüte - verströmt verlockenden Duft - zeigt ihre Strahlkraft - schliesst und schützt nachts ihre Blüte - wiegt sich sanft auf den Wellen - attraktiver Blickfänger - sattes Grün - natürlich - feine, weiche Blütenblätter - leuchtet | - shining - standing in the centre - I show my beauty - blooming - growing in the light - both strong and delicate - the most beautiful far and wide - new shoots - leaf as protection for the bud - sweet perfume - joyful opening to the sun - deeply rooted, floating on the surface - lotus-friendly waters - growth - transparency - in the midst of many - in full bloom - emits a seductive scent - shows its radiance - closes and protects its flower at night - floats gently on the waves - attractive eye-catcher - lush green - natural - fine, soft flower petals - glows |
| Picture of a hiker | - ich gehe meinen Weg - Schritt für Schritt voran - im eigenen Tempo - gut gerüstet - der Berg ruft! - frische Bergluft - Orientierung haben - rhythmische Schritte - Herausforderungen machen Spass - im Vertrauen auf die Stärke des Körpers - über Berg und Tal - neuen Zielen entgegen - Aufbruch - die Steine unter den Sohlen knirschen - guten Mutes unterwegs sein - Kräuterduft am Wegesrand - Wandern - Bergpanorama - die eigene Kraft spüren - Freude am Laufen - auf zum Gipfel - durch blühende Täler - mit festem Schritt - Los geht’s! - Bergführer | - on my path - step by step forward - at my own pace - well equipped - the mountain is calling! - fresh mountain air - sense of orientation - rhythmic steps - challenges are fun - trusting in the strength of my body - over hill and dale - towards new goals - pioneering spirit - the crunch of gravel under my feet - happy under way - the scent of herbs by the wayside - hiking - mountain panorama - feeling one's own strength - joy of walking - approaching the summit - through flowering valleys - with firm steps - let's go! - mountain guide |

*Note.* Due to copyright reasons the pictures cannot be displayed. The pictures are taken from *Ressourcen aktivieren mit dem Unbewussten: Manual und ZRM-Bildkartei [Activating resources with the unconscious: manual and ZRM picture inventory]*, by F. Krause and M. Storch, 2010, Bern: Huber.

# Instructions of the Distraction Condition

| German (original) | English (translation) |
| --- | --- |
| Schreiben Sie bitte den unten stehenden Text ab. Klicken Sie bitte erst auf "weiter", wenn Sie den Text vollständig abgeschrieben haben (der "weiter" Button wird nach einiger Zeit eingeblendet).  **Text:** 1606 legte Kurfürst Friedrich IV. von der Pfalz den Grundstein zum Bau der Zitadelle Friedrichsburg und beauftragte den holländischen Festungsarchitekten Bartel Janson die Stadt zu erweitern. Die damalige Planung eines gitterförmigen Straßennetzes für die mit der Festung verbundene Bürgerstadt Mannheim ist bis heute erhalten geblieben. Auf diese in etwa gleich großen Baublöcke ist die Bezeichnung Quadratestadt zurückzuführen. Am 24. Januar 1607 erhielt Mannheim von Kurfürst Friedrich IV. die Stadtprivilegien. 1652 verlieh Kurfürst Karl Ludwig von der Pfalz erweiterte Stadtprivilegien, um den Wiederaufbau zu begünstigen. 1692 errichten auf dem rechten Neckarufer zurückgekehrte Bürger die Siedlung Neu-Mannheim. Kurfürst Johann Wilhelm forderte zum Wiederaufbau der Stadt auf. Um die geflohenen Bürger zur Rückkehr zu bewegen und neue Zuwanderer anzuziehen, erließ der Kurfürst 1698 nochmals erweiterte Privilegien. 1709 wurde die Festung Friedrichsburg mit der Stadt Mannheim vereinigt. | Please copy the text below. Please do not click on "continue" until you have completely transcribed the text (the "continue" button will appear after a while).  **Text:** In 1606, Friedrich IV elector of Palatinate laid the foundation stone for the construction of the Friedrichsburg citadel and commissioned the Dutch fortress architect Bartel Janson to expand the city. The planning at that time of a grid-shaped road network for the bourgeois city of Mannheim, which is connected to the fortress, has been preserved to this day. The term "square city" can be traced back to these building blocks of approximately the same size. On January 24, 1607, Mannheim was granted city privileges by elector Friedrich IV. In 1652, Karl Ludwig elector of Palatinate granted extended city privileges to encourage reconstruction. In 1692 citizens who had returned to the city built the Neu-Mannheim settlement on the right bank of the Neckar. Elector Johann Wilhelm called for the reconstruction of the town. In order to persuade the fled citizens to return and to attract new immigrants, the elector issued further extended privileges in 1698. In 1709 the Friedrichsburg Fortress was united with the city of Mannheim. |

# Reliability Analysis Feelings of Autonomy Scale in Study 1 at Time 1

| Cronbach's alpha | Number of items |  |
| --- | --- | --- |
| 0,650 | 4 |  |
|  |  |  |
| Instructions and items in German/English |  |  |
|  | | |
| Wenn ich meine Pflicht angehe, dann.../ When I work on my obligation, … | corrected item-total correlation | Cronbach's alpha if item deleted |
| ...fühle ich mich gezwungen./ I feel forced. (reverse coded) | 0,421 | 0,595 |
| ...muss ich mich selbst zwingen./ I have to force myself. (reverse coded) | 0,391 | 0,608 |
| ...fühle ich mich frei./ I feel free. | 0,467 | 0,574 |
| ...kann ich so sein, wie ich bin./ I can be myself. | 0,476 | 0,549 |
|  |  |  |

# Study 1 – exploratory measures and results

**Measures.**

*Feelings when thinking of the obligation**.* To potentially learn more about the unpleasant obligations, we asked participants directly after writing down their unpleasant obligation at the beginning of the study the open questions „How do you feel when you think of this obligation?” and “Why do you feel that way?”.

*Task meaningfulness.* To measure how meaningful participants perceived their obligation we used a 4-item measure consisting of items adapted from the meaningful activity participation assessment (MAPA; Eakman, Carlson, & Clark, 2010). While the aim of the MAPA is to measure the meaningfulness of 28 predefined activities and then calculate an overall meaningfulness score, our aim was to measure meaningfulness for just one activity — the unpleasant obligation. We used the wording of the original measure and transformed it into three semantic differential scales. In the final scale, participants indicated how meaningful they perceived their obligation on 8-point semantic differential scales following the statement “I consider my obligation to be...”. The endpoints of the scales were: *meaningless* - *very meaningful*; *of no relevance* - *of high relevance*; *not personally fulfilling* - *personally fulfilling*. We averaged the items to yield a total score of task meaningfulness (Cronbach’s a = .79 - .84).

*Motivating and soothing effects.* To measure to which extent participants in the motto-goal condition experienced the motto-goal as having a motivating or soothing effect we created two 1-item measures consisting of adapted items from the volitional components questionnaire’s self-motivation and self-soothing subscales (Fröhlich & Kuhl, 2003; Kuhl & Fuhrmann, 1998). The final items were “The motto has a soothing effect (it helps to reduce nervousness or to loosen tension).” and “The motto has a motivating effect (it helps to increase pleasure or to foster awareness of the positive aspects of the obligation).” Participants indicated their agreement to the items on 7-point scales ranging from 1 (*no agreement*) to 7 (*full agreement*). We chose these two single item measurements to ensure economical assessment, while ensuring high face validity of our items.

**Results.**

*Feelings when thinking of the obligation.* In reply to the question how they feel participants wrote on average 7.06 words (*SD* = 8.04) and in reply to the question why they feel that way 17.43 (*SD* = 12.03) words. In general, the answers reflect that participants wrote about unpleasant and important obligations. We did not further analyze the content of answers.

*Task meaningfulness.* A mixed GLM with Time (Time 1 vs. Time 2) as a within factor and Condition (motto-goal vs. distraction) as a between factor yielded a significant main effect of Time, *F*(1, 119) = 8.17, *p* = .005, η_p_^2^ = .064, indicating a significant in- crease in task meaningfulness from Time 1 to Time 2 independent of condition. A significant interaction effect between Time and Condition emerged, *F*(1, 119) = 7.40, *p* = .007, η_p_^2^ = .059. This effect indicates that the increase in task meaningfulness differed in the conditions. Paired t-tests were performed, comparing the simple main effect for each condition: In the motto-goal condition there was a significant increase in ratings of task meaningfulness from Time 1 (*M* = 5.37, *SD* = 1.73) to Time 2 (*M* = 5.81, *SD* = 1.78), *t*(60) = 4.23, *p* < .001, *d* = 0.25. In the distraction condition there was no significant change in task meaningfulness from Time 1 (*M* = 5.56, *SD* = 1.62) to Time 2 (*M* = 5.57, *SD* = 1.87), *t*(59) = 0.92, *p* = .93, *d* = 0.06.

*Motivating and soothing effects.* To gain first insights into the question whether motto-goals are experienced by participants as having a motivating or a soothing effect, we looked at the distribution of motivating and soothing ratings. To categorize motto-goals in motivating vs. not motivating and soothing vs. not soothing, we chose 3.5 as a cut-off criterion (scale ranges from 1 to 7), as the label for 4 was “somewhat true”, representing somewhat motivating or soothing motto-goals, respectively. Under this definition, 6.6% of participants experienced the motto-goal as neither motivating nor soothing, 8.2% of participants experienced the motto-goal as soothing but not motivating, 14.8% experienced the motto-goal as motivating but not soothing and the majority of 78.7% experienced the motto-goal as both motivating and soothing.

**Interpretation.**

The answers to the open questions asked directly after writing down their unpleasant obligation indicated that participants wrote about unpleasant and important obligations. This supports the results of our main measures of affective and cognitive attitude towards the obligation. Answering the open questions implies that participants spend extensive thought about how they felt when thinking of their obligation. Since the open questions were asked at the beginning of the study before baseline measurement and before condition manipulation to all participants, we do not think answering the questions affected differences found between conditions.

Exploratory analyses indicated a significant increase in task meaningfulness. The initial values of

task meaningfulness were rather high (5.5 on a scale from 1 to 7) and could indicate that — in the context of tasks which are judged as unpleasant and important — the judgement as important entails task meaningfulness. Although the effect size was small in comparison to the other effects, the results suggest that personally meaningful content is also activated by applying a motto-goal. In the future, task meaningfulness would be an interesting construct to take into consideration when studying the effect of motto-goals in contexts other than unpleasant obligations.

In order to shed further light on motto-goals, we attempted to categorize motto-goals as motivating *or* soothing. The results show that while some participants experience their motto-goal as motivating or soothing, the majority of participants experienced their motto-goal as motivating *and* soothing at the same time.

# Study 2 – exploratory measures and results

**Measures.**

*Feelings when thinking of the obligation.* To potentially learn more about the unpleasant obligations, we asked participants directly after writing down their unpleasant obligation at the beginning of the study the open questions „How do you feel when you think of this obligation?” and “Why do you feel that way?”.

*Self-efficacy.* To measure self-efficacy regarding the obligation we used a 3-item scale from Pomaki, Maes, & ter Deust (2004). The scale was translated into German by Dargel (2005). The original scale measures goal specific self-efficacy; we adapted it to measure obligation specific self-efficacy (e.g. “I possess the necessary skills to achieve this goal.” was changed into “I possess the necessary skills to fulfill this obligation.”). Responses were given on a 5-point scale from 1 (*no agreement)* to 5 (*full agreement*) and averaged to yield a total score (Cronbach’s α = .77).

**Results.**

*Feelings when thinking of the obligation.* In reply to the question how they feel participants wrote on average 6.44 words (*SD* = 7.58) and in reply to the question why they feel that way 16.48 (*SD* = 12.74) words. In general, the answers reflect that participants wrote about unpleasant and important obligations. We did not further analyze the content of answers.

*Self-efficacy.* A one-way ANOVA yielded a non-significant tendency pattern of differences across conditions, *F*(2, 128) = 2.18, *p* = .11, η_p_^2^ = .033. Using the same contrasts as in the main analysis revealed higher self-efficacy ratings in the motto-goal condition (*M* = 3.87, *SD* = 0.90) compared to the distraction condition (*M* = 3.50, *SD* = 0.89), *t*(128) = 2.00 , *p* = .048, *d* = 0.28; however, participants in the motto-goal condition did not report significantly higher self-efficacy ratings compared to participants in the placebo condition (*M* = 3.83, *SD* = 0.83), *t*(128) = 0.25, *p* = .81, *d* = 0.03. Post-hoc analyses using Dunnett-T indicated a non-significant trend that the participants in the placebo condition had higher ratings on self-efficacy than the distraction condition (*p* = .17).

**Interpretation**

The answers to the open questions asked directly after writing down their unpleasant obligation indicated that participants wrote about unpleasant and important obligations. This supports the results of our main measures of affective and cognitive attitude towards the obligation. Answering the open questions implies that participants spend extensive thought about how they felt when thinking of their obligation. Since the open questions were asked at the beginning of the study before initial measurement (of affective and cognitive attitude) and before condition manipulation to all participants, we do not think answering the questions affected differences found between conditions.

Exploratory analyses indicated that participants in the motto-goal condition reported higher perceived self-efficacy compared to the distraction condition but showed no advantage compared to the placebo condition. This could be an indicator that both interventions are effective at raising self-efficacy levels, probably through demand characteristics and general positive expectancy (Haaga & Stiles, 2000). Nevertheless, the effect size was small, and post-hoc analyses revealed that participants in the placebo condition merely showed a tendency to report higher self-efficacy ratings compared to participants in the distraction condition (*p* = .17).
